# Recurrent Circuitry for Balancing Sleep Need and Sleep

**DOI:** 10.1016/j.neuron.2017.12.016

**Published:** 2018-01-17

**Authors:** Jeffrey M. Donlea, Diogo Pimentel, Clifford B. Talbot, Anissa Kempf, Jaison J. Omoto, Volker Hartenstein, Gero Miesenböck

**Affiliations:** 1 Centre for Neural Circuits and Behaviour, University of Oxford, Tinsley Building, Mansfield Road, Oxford OX1 3SR, UK; 2 Department of Neurobiology, David Geffen School of Medicine at the University of California, Los Angeles, Los Angeles, CA 90095-1763, USA; 3 Department of Molecular, Cell and Developmental Biology, University of California, Los Angeles, Los Angeles, CA 90095-1606, USA

**Keywords:** sleep homeostasis, sleep pressure, arousal, *Drosophila* melanogaster, central complex, fan-shaped body, ellipsoid body, relaxation oscillator

## Abstract

Sleep-promoting neurons in the dorsal fan-shaped body (dFB) of *Drosophila* are integral to sleep homeostasis, but how these cells impose sleep on the organism is unknown. We report that dFB neurons communicate via inhibitory transmitters, including allatostatin-A (AstA), with interneurons connecting the superior arch with the ellipsoid body of the central complex. These “helicon cells” express the galanin receptor homolog AstA-R1, respond to visual input, gate locomotion, and are inhibited by AstA, suggesting that dFB neurons promote rest by suppressing visually guided movement. Sleep changes caused by enhanced or diminished allatostatinergic transmission from dFB neurons and by inhibition or optogenetic stimulation of helicon cells support this notion. Helicon cells provide excitation to R2 neurons of the ellipsoid body, whose activity-dependent plasticity signals rising sleep pressure to the dFB. By virtue of this autoregulatory loop, dFB-mediated inhibition interrupts processes that incur a sleep debt, allowing restorative sleep to rebalance the books.

**Video Abstract:**

## Highlights

• Sleep-promoting dFB neurons inhibit helicon cells of the central complex
• Helicon cells transmit visual signals to R2 ring neurons and gate locomotion
• Neurons generating sleep need and sleep-inducing neurons are recurrently connected
• A unified mechanism accounts for sensory, motor, and homeostatic features of sleep

## Teaser

Neurons encoding sleep need and sleep-inducing neurons are recurrently connected. A crucial link in the circuit is necessary for visually guided movements but inhibited during sleep. A unified mechanism can thus account for sensory, motor, and homeostatic aspects of sleep.

## Introduction

The behavioral hallmarks of sleep are manifold. They include inactivity, reduced responsiveness to external stimuli, rapid reversibility, and homeostatic rebound after sleep loss. Any sleep control system must therefore fulfill a multitude of functions—blocking locomotor activity, gating sensory pathways, inhibiting arousal systems, relieving sleep pressure—and perhaps also directly influence processes germane to a fundamental purpose of sleep, be it metabolic recovery (Vyazovskiy and Harris, 2013, Walker et al., 1979), memory consolidation (Wilson and McNaughton, 1994), or synaptic scaling (Tononi and Cirelli, 2003).

Surprisingly, given these diverse and widespread manifestations, activity in a tiny minority of two dozen neurons (of a total of ∼100,000 in the brain) suffices to induce sleep in *Drosophila* (Donlea et al., 2011). The sleep-promoting neurons send projections to the dorsal fan-shaped body (dFB) of the central complex and act as a feedback controller or homeostat (Donlea et al., 2014). Their operating principle is remarkably simple: sleep need is encoded in the intrinsic electrical excitability of the sleep-inducing cells, which fluctuates because two potassium conductances, voltage-gated Shaker and the leak channel Sandman, are modulated antagonistically (Donlea et al., 2014, Pimentel et al., 2016). As sleep pressure builds during waking, the sleep-promoting neurons switch from electrical silence to activity and the animal from wakefulness to restorative sleep. The self-correcting nature of feedback is thus embodied in the biophysics of an excitability switch.

dFB neurons can be arrested in the electrically silent state by mutating the Rho-GTPase-activating protein Crossveinless-c (Cv-c) (Donlea et al., 2014). The mutation likely prevents the internalization of Sandman that is a prerequisite for flipping the neurons’ sleep-promoting activity back on (Pimentel et al., 2016). *cv-c* mutants suffer profound insomnia (along with its cognitive consequences) and are unable to sense and/or correct sleep deficits (Donlea et al., 2014). In contrast to our growing understanding of the sleep-control neurons themselves, however, neither the signals released by them to induce sleep, nor any of their downstream targets, nor the manner in which they regulate these targets have been identified.

Among the many sleep-regulatory structures in mammals (for reviews, see Brown et al., 2012, Saper et al., 2010, Weber and Dan, 2016), a cluster of sleep-active neurons in the ventrolateral preoptic nucleus (VLPO) of the hypothalamus exhibit perhaps the clearest parallels with dFB neurons in flies. VLPO activation is tightly correlated with sleep (Kaitin, 1984, Sherin et al., 1996, Szymusiak et al., 1998, Takahashi et al., 2009), and VLPO lesions fracture the sleep-wake cycle, producing insomnia (Lu et al., 2000). Like dFB neurons, VLPO neurons modulate their firing rates according to sleep need, with activity peaking at the beginning of recovery sleep (Alam et al., 2014, Szymusiak et al., 1998, Takahashi et al., 2009). VLPO neurons secrete the inhibitory neuropeptide galanin along with the classical inhibitory transmitter GABA (Sherin et al., 1998) and project to the tuberomamillary nucleus and other arousal centers in the brain stem (Hsieh et al., 2011, Sherin et al., 1998, Steininger et al., 2001), which often form reciprocal inhibitory connections with the VLPO (Chou et al., 2002). Mutual antagonism between neurons promoting sleep and waking thus creates a bistable flip-flop arrangement (Saper et al., 2010, Saper et al., 2005). Projections from VLPO neurons to structures other than arousal centers have not been described, leaving open the question of whether sleep-promoting cells can directly control motor or sensory pathways or whether they do so only indirectly by inhibiting arousal systems.

Here we begin to explore the circuitry downstream of sleep-control neurons in *Drosophila*. We find that dFB neurons induce sleep via a range of inhibitory transmitters that include the neuropeptide allatostatin-A (AstA). Among the targets of AstA are a group of interneurons of the central complex that we term helicon cells. These neurons are inhibited by sleep-promoting AstA, excited by visual input, permissive for locomotion, and presynaptic to R2 ring neurons of the ellipsoid body, whose activity has been linked to the accumulation of sleep debt (Liu et al., 2016). dFB-mediated inhibition of helicon cells may thus account for three cardinal features of sleep: elevated visual thresholds, immobility, and the dissipation of sleep need.

## Results

### A Sleep-Promoting Signal from dFB Neurons

**Figure 1:**
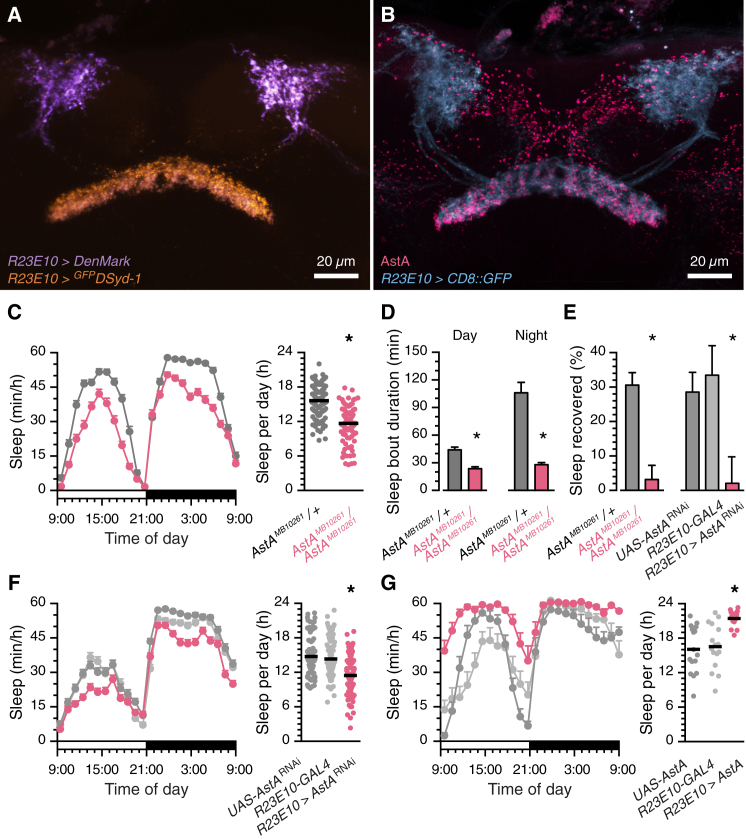
dFB Neurons Regulate Sleep via AstA (A) *R23E10-GAL4*-driven expression of the presynaptic marker ^GFP^DSyd-1 (orange) and the dendritic marker DenMark (magenta) in dFB neurons. Axon terminals are concentrated in one layer of the dFB; dendrites extend into the dorsal protocerebrum. (B) AstA immunostaining (red) overlaps with the axonal projections of sleep-promoting dFB neurons expressing *R23E10-GAL4*-driven CD8::GFP (blue). (C) Homozygous *AstA*^*MB10261*^ mutants (red) sleep less than heterozygous controls (gray) during the course of a 24-hr day (left, mean ± SEM, n = 91–113 flies per group). White and black bars denote periods of light and darkness, respectively. Two-way repeated-measures ANOVA of the hour-by-hour sleep time course detects a significant genotype × time interaction (p < 0.0001). Total sleep is reduced by ∼25% in *AstA*^*MB10261*^ mutants compared with heterozygous controls (p < 0.0001, Mann-Whitney test). Circles symbolize individual flies; horizontal lines indicate group means. (D) Homozygous *AstA*^*MB10261*^ mutants (red) exhibit shorter sleep bouts than heterozygous controls (gray) during the day (left, mean ± SEM; p < 0.0001, Mann-Whitney test) and night (right, mean ± SEM; p < 0.0001, Mann-Whitney test). (E) After overnight sleep deprivation for 12 hr, homozygous *AstA*^*MB10261*^ mutants (red, left, mean ± SEM, n = 121–124 flies per group) and flies expressing *AstA*^RNAi^ under the control of *R23E10-GAL4* (red, right, mean ± SEM, n = 51–55 flies per group) show a reduced sleep rebound relative to heterozygous *AstA*^*MB10261*^ mutants or parental controls, respectively (left: p < 0.0001, Mann-Whitney test; right: p = 0.0189, Kruskal-Wallis ANOVA). The asterisk on the right denotes a significant difference from both parental controls in pairwise *post hoc* comparisons. (F) Expression of *AstA*^RNAi^ under the control of *R23E10-GAL4* (red) reduces sleep compared with parental controls (light gray, *R23E10-GAL4*/+; dark gray, *UAS-AstA*^RNAi^/+) (mean ± SEM, n = 31–34 flies per group). White and black bars denote periods of light and darkness, respectively. Two-way repeated-measures ANOVA of the hour-by-hour sleep time course detects a significant genotype × time interaction (left, p < 0.0001); one-way ANOVA detects a significant genotype effect on total sleep time (right, p < 0.0001). Circles symbolize individual flies; horizontal lines indicate group means. The asterisk denotes a significant difference from both parental controls in pairwise *post hoc* comparisons. (G) Overexpression of AstA under the control of *R23E10-GAL4* (red) increases sleep compared with parental controls (light gray, *R23E10-GAL4*/+; dark gray, *UAS-AstA*/+) (mean ± SEM, n = 14–16 flies per group). White and black bars denote periods of light and darkness, respectively. Two-way repeated-measures ANOVA of the hour-by-hour sleep time course detects a significant genotype × time interaction (left, p < 0.0001); one-way ANOVA detects a significant genotype effect on total sleep time (right, p < 0.0001). Circles symbolize individual flies; horizontal lines indicate group means. The asterisk denotes a significant difference from both parental controls in pairwise *post hoc* comparisons. See also Figure S1.

Sleep-promoting neurons marked by *R23E10-GAL4* (Donlea et al., 2014, Jenett et al., 2012) project their axons to a single dorsal stratum of the fan-shaped body, where they form numerous synaptic release sites revealed by decoration with ^GFP^DSyd-1 (Owald et al., 2010; Figure 1A). Of the at least eight neuropeptides detected in different layers or layer combinations of the fan-shaped body (Kahsai and Winther, 2011), the distribution of AstA partially overlaps the axons of *R23E10-GAL4*-positive neurons (Figure 1B), hinting that sleep-promoting neurons may be a source of AstA. To corroborate this notion, we examined AstA immunoreactivity and sleep in carriers of *AstA*^*MB10261*^, a transposon insertion in the 3′ UTR of the *AstA* locus that disrupts the *AstA-RA* isoform (Figure S1A). Homozygous carriers of *AstA*^*MB10261*^ lacked detectable AstA staining in the dFB (but retained some AstA immunoreactivity elsewhere in the brain; Figures S1B and S1C) and slept ∼25% less than heterozygous controls (Figures 1C and S1D) because of shorter sleep episodes during the day and night (Figure 1D). This sleep maintenance insomnia is reminiscent of that of *cv-c* mutants (Donlea et al., 2014), in whom uncontrolled cell surface expression of Sandman is thought to short-circuit the spike generator of dFB neurons (Pimentel et al., 2016). Like *cv-c* mutants, *AstA* mutants exhibited robust free-running circadian rhythms after entrainment (Figure S1E) but failed to compensate homeostatically for a night of mechanical sleep deprivation (Figure 1E). Similar mutant phenotypes are, of course, expected if some of the action potential output of dFB neurons is conveyed by AstA.

Despite these similarities, the sleep disruptions of *AstA*^*MB10261*^ homozygotes were milder than those of *cv-c* transheterozygotes (Donlea et al., 2014), leaving scope for GABAergic or peptidergic cotransmission, as in VLPO neurons (Sherin et al., 1998), or transmitter heterogeneity within the dFB neuron population. Indeed, the vesicular GABA transporter (vGAT) and several neuropeptides in addition to AstA (Kahsai and Winther, 2011) were translated by *R23E10-GAL4*-positive neurons (Figure S1G), and spatially restricted RNA-mediated interference (RNAi) with the expression of some of these peptides (e.g., myoinhibiting peptide) implicated them, too, in the regulation of sleep (Figure S1H). While AstA is thus unlikely the only sleep-promoting signal released by dFB neurons, it remains our sole focus here.

To tie the role of AstA in homeostatic sleep control to dFB neurons, we altered AstA levels selectively in these cells. RNAi knockdown, using either the exquisitely dFB-specific *R23E10-GAL4* driver (Donlea et al., 2014) or the somewhat broader *104y-GAL4* line (Donlea et al., 2011, Rodan et al., 2002, Sakai and Kitamoto, 2006), reduced basal sleep relative to parental controls (Figures 1F, S1D, S1F, and S1I) and eliminated the homeostatic response to sleep deprivation (Figure 1E). dFB-restricted overexpression of a transgene encoding *AstA*, again with the help of the *R23E10-GAL4* and *104y-GAL4* drivers, had the opposite effect; it elevated sleep time (Figure 1G; Figure S1J).

### Helicon Cells: Targets of dFB Neurons with Projections to the Ellipsoid Body

**Figure 2:**
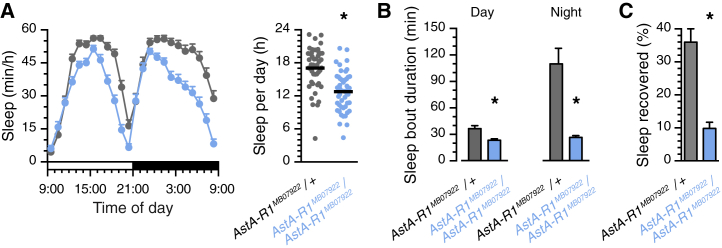
A Mutation in *AstA-R1* Reduces Sleep (A) Homozygous *AstA-R1*^*MB07922*^ mutants (blue) sleep less than heterozygous controls (gray) during the course of a 24-hr day (left, mean ± SEM, n = 46–48 flies per group). White and black bars denote periods of light and darkness, respectively. Two-way repeated-measures ANOVA of the hour-by-hour sleep time course detects a significant genotype × time interaction (p < 0.0001). Total sleep is reduced by ∼25% in *AstA-R1*^*MB07922*^ mutants compared with heterozygous controls (p < 0.0001, Mann-Whitney test). Circles symbolize individual flies; horizontal lines indicate group means. (B) Homozygous *AstA-R1*^*MB07922*^ mutants (blue) exhibit shorter sleep bouts than heterozygous controls (gray) during the day (left, mean ± SEM; p = 0.0012, Mann-Whitney test) and night (right, mean ± SEM; p < 0.0001, Mann-Whitney test). (C) After overnight sleep deprivation for 12 hr, homozygous *AstA-R1*^*MB07922*^ mutants (blue) show a reduced sleep rebound relative to heterozygous controls (gray) (mean ± SEM, n = 107–112 flies per group; p < 0.0001, Mann-Whitney test).

Knowledge of a neuropeptide secreted by dFB neurons allowed us to search for postsynaptic targets among neurons expressing AstA receptors (AstA-Rs). To pinpoint the relevant receptor type(s), we measured sleep in flies carrying mutant *AstA-R* alleles. Flies homozygous for *AstA-R1*^*MB07922*^, a transposon insertion in the *AstA-R1* locus, exhibited a short-sleeping phenotype that mirrored that of flies lacking AstA, suggesting a match between receptor and ligand: like homozygous *AstA*^*MB10261*^ mutants, homozygous *AstA-R1*^*MB07922*^ mutants lost ∼25% of their daily sleep compared to heterozygous controls (Figures 2A and S2A); as in *AstA* mutants, the loss in overall sleep time was caused by a shortening of sleep bouts during the day and night (Figure 2B) and accompanied by reduced rebound sleep after a night of enforced sleeplessness (Figure 2C).

**Figure 3:**
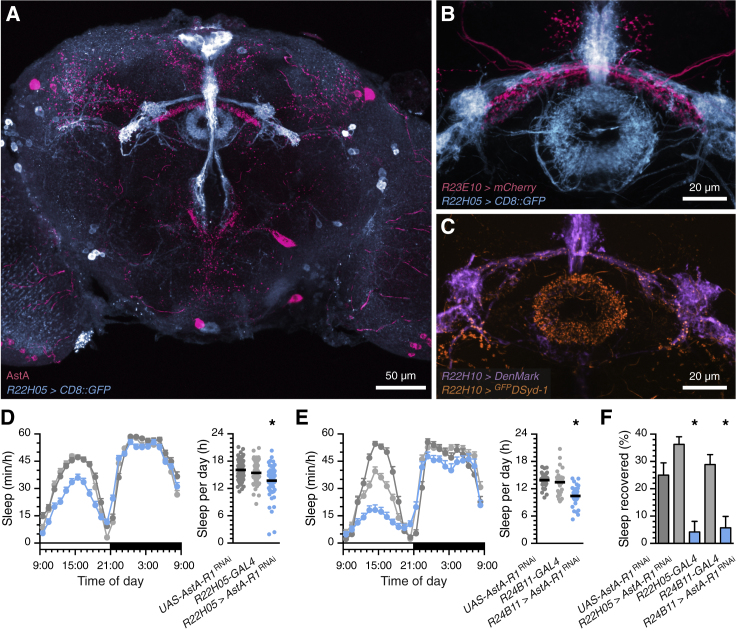
AstA-R1 Functions in Helicon Cells to Regulate Sleep (A) The *AstA-R1* enhancer element in *R22H05-GAL4* drives transgene expression in a small number of neurons in the brain (blue), which include four cells whose neurites contact AstA-immunopositive puncta in the dFB (red). (B) Central complex neurons labeled by *R22H05-GAL4* (blue) connect the superior arch to the ellipsoid body via fibers that pass through the dFB. The connecting fibers closely adjoin sleep-promoting dFB neurons marked by *R23E10-LexA* (red). (C) *R22H05-GAL4*-driven expression of the presynaptic marker ^GFP^DSyd-1 (orange) and the dendritic marker DenMark (magenta) in helicon cells. Axon terminals are concentrated in the bulb and in rings of the ellipsoid body; dendrites extend into the superior arch. (D) Expression of *AstA-R1*^RNAi^ under the control of *R22H05-GAL4* (blue) reduces sleep compared with parental controls (light gray, *R22H05-GAL4*/+; dark gray, *UAS-AstA-R1*^RNAi^/+) (mean ± SEM, n = 63–64 flies per group). White and black bars denote periods of light and darkness, respectively. Two-way repeated-measures ANOVA of the hour-by-hour sleep time course detects a significant genotype × time interaction (left, p < 0.0001); Kruskal-Wallis ANOVA detects a significant genotype effect on total sleep time (right, p < 0.0001). Circles symbolize individual flies; horizontal lines indicate group means. The asterisk denotes a significant difference from both parental controls in pairwise *post hoc* comparisons. (E) Expression of *AstA-R1*^RNAi^ under the control of *R24B11-GAL4* (blue) reduces sleep compared with parental controls (light gray, *R24B11-GAL4/+*; dark gray, *UAS-AstA-R1*^RNAi^/+) (mean ± SEM, n = 30–32 flies per group). White and black bars denote periods of light and darkness, respectively. Two-way repeated-measures ANOVA of the hour-by-hour sleep time course detects a significant genotype × time interaction (left, p < 0.0001); Kruskal-Wallis ANOVA detects a significant genotype effect on total sleep time (right, p < 0.0001). Circles symbolize individual flies; horizontal lines indicate group means. The asterisk denotes a significant difference from both parental controls in pairwise *post hoc* comparisons. (F) After overnight sleep deprivation for 12 hr, flies expressing *AstA-R1*^RNAi^ under the control of *R22H05-GAL4* or *R24B11-GAL4* show a reduced sleep rebound relative to parental controls (mean ± SEM, n = 59–64 flies per group; p < 0.0001, Kruskal-Wallis ANOVA). Asterisks denote significant differences from both parental controls in pairwise *post hoc* comparisons. See also Figures S2 and S3.

Two *GAL4* lines incorporating enhancer modules of the *AstA-R1* locus, *R22H05-GAL4* and *R22H10-GAL4* (Jenett et al., 2012), drive expression in a small number of neurons in the brain. These include a cluster of neuroendocrine cells in the *pars intercerebralis* and a handful of cells in the central complex (Figure 3A). Here, both *R22H05-GAL4* and *R22H10-GAL4* label four large interneurons that connect the superior arch to the ellipsoid body via fibers that pass near AstA-immunopositive puncta (Figure 3A) through the *R23E10-LexA*-positive layer of the dFB (Figure 3B). Because the spiral circular morphology of the individually labeled neurons (see below) resembles the brass instrument, we term these interneurons helicon cells. When expressed in helicon cells, the dendritic marker DenMark (Nicolaï et al., 2010) localized to the superior arch, while ^GFP^DSyd-1 (Owald et al., 2010) labeled presynaptic boutons in the bulb and the concentric rings of the ellipsoid body (Figure 3C). dFB neurons may thus gate the flow of signals from dendritic sites in the superior arch to axon terminals in the ellipsoid body.

Reducing AstA-R1 levels within helicon cells via *R22H05-GAL4* or *R22H10-GAL4*-driven expression of an RNAi transgene decreased the total amount of sleep (Figures 3D and S2B), with an especially pronounced effect on the length of sleep episodes during the afternoon siesta (Figures 3D and S2A–S2C). Although these are some of the expected consequences of rendering dFB targets insensitive to the sleep-promoting effect of AstA, the interpretation of this experiment is ambiguous because the expression domains of both GAL4 lines include neurons of the *pars intercerebralis* (Figure 3A), which themselves have been implicated in the regulation of sleep (Crocker et al., 2010, Foltenyi et al., 2007). To resolve this ambiguity, we used a third GAL4 driver, *R24B11-GAL4* (Jenett et al., 2012), which captured the four helicon cells and a few currently unidentified cells in the dorsal brain but spared the neuroendocrine cells (Figures S3A and S3B). *R24B11-GAL4*-driven interference with the expression of *AstA-R1* recapitulated the sleep phenotypes seen with the *R22H05-GAL4* and *R22H10-GAL4* lines (Figures 3E, 3F, and S2A–S2D). Because helicon cells are the only neuronal elements in common to all three expression patterns (Figures S3A and S3B), the observed sleep changes must reflect the loss of *AstA-R1* from them.

The consequences for sleep of depleting AstA-R1 from helicon cells differed subtly from those of removing AstA from dFB neurons and also from those of the genomic mutations: whereas RNAi-mediated interference with *AstA-R1* expression in helicon cells caused the most profound and consistent sleep loss during the day (Figures 3D, 3E, S2A and S2B), homozygous carriage of the mutant *AstA-R1*^*MB07922*^ allele and diminished allatostatinergic transmission from dFB neurons produced sleep deficits also during the night (Figures 1, 2, S1, and S2). These differences could arise if helicon cells retained some AstA-R1 after knockdown or if dFB neurons controlled AstA-responsive targets in addition to helicon cells. In our view, the dissociation between the daytime and nighttime effects of the cell-specific receptor manipulation and the fact that only a fraction of dFB neuron terminals are found in immediate proximity to helicon cell neurites (Figures 3A and 3B) favor the latter interpretation.

### dFB Neurons Inhibit Helicon Cells and Their Visual Responses

**Figure 4:**
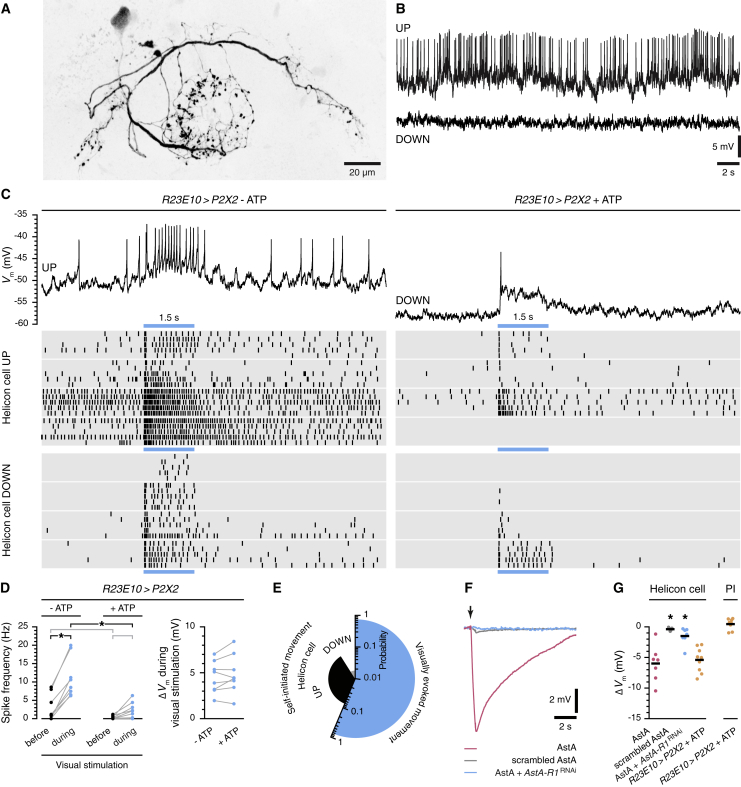
dFB Neurons Inhibit Helicon Cells and Their Visual Responses (A) Morphology of a single biocytin-filled helicon cell. (B) Membrane potential of the helicon cell shown in (A) during UP and DOWN states. (C) Responses of helicon cells to visual stimulation (blue bars, 1.5 s illumination at 450–490 nm). Top: membrane potential. Bottom: spike rasters of 8 helicon cells during five visual stimulation trials. Left: helicon cell responses before the activation of P2X2-expressing dFB neurons with ATP. Right: responses of the same 8 helicon cells after the activation of P2X2-expressing dFB neurons with ATP. (D) Visually evoked changes in spike frequency (left) and membrane potential baseline (right) before and after the activation of P2X2-expressing dFB neurons with ATP. Kruskal-Wallis ANOVA detects a significant difference in visually evoked spike frequency changes between groups (p = 0.0003). Asterisks indicate significant differences in planned pairwise *post hoc* comparisons (black brackets); gray brackets denote pairwise comparisons without significant differences. Paired t test fails to detect a significant difference in visually evoked changes in membrane potential baseline (p = 0.2045). (E) Frequency (slice angle) and probability (slice radius) of locomotor bouts as a function of helicon cell activity (n = 13 cells). Ninety-four bouts were visually evoked and 73 were self-initiated; of the self-initiated bouts, 58 occurred during UP states and 15 during DOWN states. The probability of a visual stimulus to elicit a locomotor bout was 0.7234; the probability of self-initiated movement was 0.0707/s during UP and 0.0179/s during DOWN states. Note that probabilities are plotted on a logarithmic scale. (F) Membrane potentials of helicon cells following the application of AstA (red) or a peptide with a scrambled AstA sequence (gray) or following the application of AstA to helicon cells expressing *AstA-R1*^RNAi^ under the control of *R22H05-GAL4* (blue). Traces are averages of 20 peptide applications. (G) Hyperpolarizations evoked by AstA or by the activation of P2X2-expressing dFB neurons with ATP. Left: recordings from helicon cells. Right: recordings from neuroendocrine cells in the *pars intercerebralis* (PI). Circles symbolize average responses of individual cells to 20 peptide applications (n = 7–8 cells per condition); horizontal lines indicate group means. Kruskal-Wallis ANOVA detects a significant difference between groups (p < 0.0001); asterisks indicate significant differences from control conditions in pairwise *post hoc* comparisons.

In whole-cell patch-clamp recordings from head-fixed flies walking or resting on an air-supported trackball, helicon cells (Figure 4A) were found in one of two states: a DOWN state characterized by the near absence of spikes (firing rate < 1 Hz) and an UP state in which the neurons fired persistently, with occasionally metronomic precision, at rates of 16.9 ± 3.6 Hz (Figures 4B and 4C). An average voltage difference of 10.9 ± 2.3 mV (mean ± SEM, n = 10 cells) separated the membrane potential baselines of the two states. Visual stimuli evoked large depolarizations, which, especially in the UP state, released intense flurries of action potentials (Figures 4C and 4D); in 68 of 94 cases (72.3%), these volleys of activity were associated with a locomotor bout (Figure 4E). Spontaneous movements were initiated with approximately 4-fold higher probability when the recorded cell was in the UP rather than in the DOWN state (Figure 4E). Together, these results suggest that helicon cells play a permissive role in visually guided movement.

*AstA-R1* encodes a G-protein-coupled receptor with homology to mammalian galanin receptors (Birgül et al., 1999, Chen et al., 2016), suggesting that sleep-regulatory signals are conserved at the receptor level (Sherin et al., 1998). Like galanin receptors (Smith et al., 1998), AstA-R1 controls the gating of G-protein-coupled potassium channels (Birgül et al., 1999, Lechner et al., 2002), whose opening in the presence of AstA is expected to inhibit AstA-R1-positive neurons. Indeed, focal pressure ejection of synthetic AstA from a pipette positioned near helicon cell neurites in the dFB hyperpolarized the cells, whereas the administration of a peptide containing the same amino acid residues in a randomly scrambled sequence, or of AstA to cells depleted of AstA-R1, elicited no response (Figures 4F and 4G).

To verify that dFB neurons are the physiological source of inhibition, we expressed the ATP-gated cation channel P2X2 (Lima and Miesenböck, 2005) under *R23E10-LexA* control and pressure-ejected 500 μM ATP onto the dendrites of dFB neurons. The simultaneously recorded membrane potentials of helicon cells, which were marked by *R22H10-GAL4*-driven GFP expression, hyperpolarized as deeply in response to the genetically targeted activation of dFB neurons as they did to the direct delivery of AstA (Figures 4C and 4G). Helicon cells fell silent or fired only sparsely during periods of evoked dFB neuron activity; of four cells found in the UP state before the application of ATP, three switched to the hyperpolarized DOWN state afterward, and all neurons initially in the DOWN state remained (Figure 4C). Although visual stimuli continued to elicit large subthreshold depolarizations in the presence of ATP, spiking responses were attenuated or abolished (Figures 4C and 4D). dFB neurons thus mute the output of helicon cells by pulling their membrane potentials away from action potential threshold.

In contrast to the profound inhibition of helicon cells, neuroendocrine cells in the *pars intercerebralis* showed no trace of modulation during artificially evoked dFB neuron activity (Figure 4G), reinforcing our conclusion that helicon cells are the sole dFB targets among the two groups of neurons marked by the *R22H05-GAL4* and *R22H10-GAL4* drivers.

### Helicon Cells Gate Locomotion

**Figure 5:**
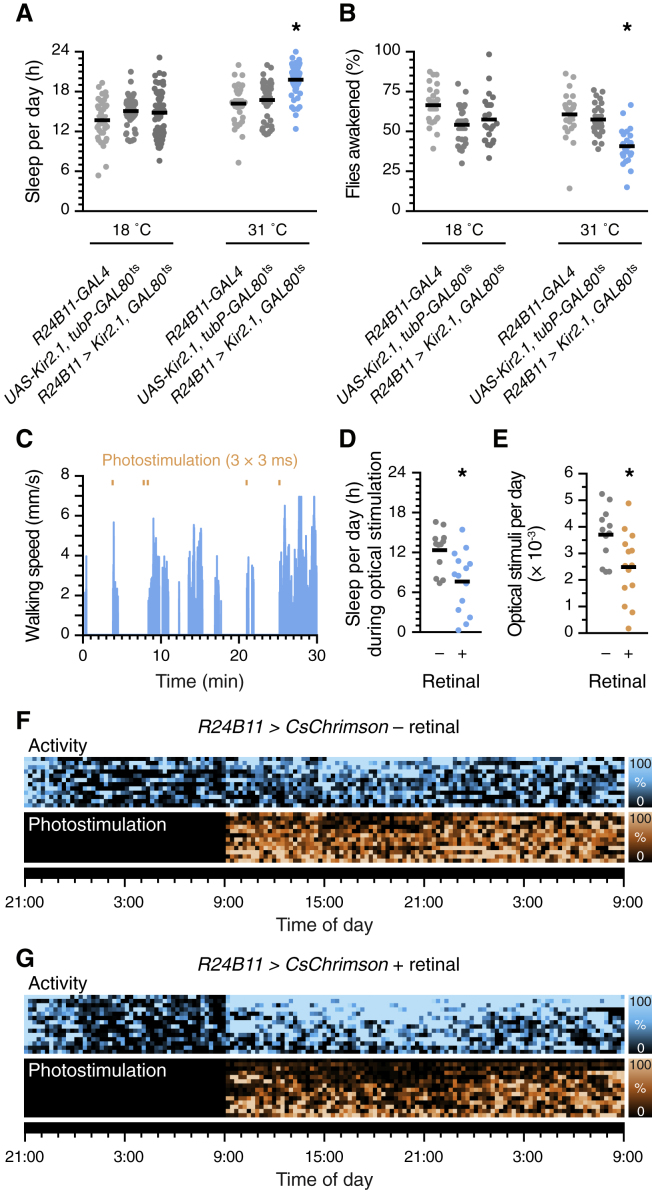
Helicon Cells Gate Locomotion (A) Temperature-inducible expression of Kir2.1 under the control of *R24B11-GAL4* increases sleep (n = 32–62 flies per group). Circles symbolize individual flies; horizontal lines indicate group means. Two-way repeated-measures ANOVA detects a significant genotype × temperature interaction (p < 0.0001); the asterisk indicates a significant difference from both parental controls in pairwise *post hoc* comparisons. (B) Temperature-inducible expression of Kir2.1 under the control of *R24B11-GAL4* reduces the percentage of flies awakened by a visual stimulus (n = 22–23 flies per group). Circles symbolize individual flies; horizontal lines indicate group means. Two-way ANOVA detects a significant genotype × temperature interaction (p = 0.0024); the asterisk indicates a significant difference from both parental controls in pairwise *post hoc* comparisons. (C) Closed-loop optogenetic control of helicon cell activity. The walking speed of a fly (blue) is continuously monitored, and photostimulation (orange) is triggered after > 3 min of inactivity. Each stimulation block consists of three optical pulses that are repeated every 30 s until the next movement occurs. (D) Inactivity-triggered photostimulation decreases sleep in retinal-fed flies expressing CsChrimson under *R24B11-GAL4* control (blue, n = 14 flies) relative to vehicle-treated controls (gray, n = 12 flies) (p = 0.0066, t test). Circles symbolize individual flies; horizontal lines indicate group means. (E) Retinal-fed flies expressing CsChrimson under *R24B11-GAL4* control (orange, n = 14 flies) receive fewer optical stimuli than vehicle-treated controls (gray, n = 12 flies) (p = 0.0154, t test). Circles symbolize individual flies; horizontal lines indicate group means. (F and G) Locomotor activity (blue, top) and exposure to photostimulation (orange, bottom) of 12 vehicle-treated (F) and 14 retinal-fed flies (G) expressing CsChrimson under *R24B11-GAL4* control. Individuals in each group are sorted in descending order of locomotor activity during photostimulation. Matching rows in the activity and photostimulation plots report simultaneously logged data from the same individual. Colored squares represent 15-min time bins. Within each bin, the percentages of time spent moving or exposed to photostimulation are color-coded according to the look-up tables on the right. The 24-hr experimental period is preceded by a night of baseline sleep. Stimulation light pulses notwithstanding, the animals were raised and kept in constant darkness.

If dFB neurons promote rest by inhibiting helicon cells, then reducing the electrical activity of these neurons should, in itself, produce behavioral inactivity. Given the prominent responses of helicon cells to light (Figure 4C), the same manipulation is also expected to raise the threshold for visually evoked locomotor bouts (Figure 4E). To test these expectations, we used *R24B11-GAL4* to place an inwardly rectifying potassium channel (Kir2.1) into helicon cells (Baines et al., 2001) and timed the expression of the conductance with the help of the temperature-sensitive repressor of GAL4, GAL80^ts^ (McGuire et al., 2003). At the permissive temperature of 18°C, when functional GAL80^ts^ prevented the expression of Kir2.1, locomotion and the percentage of inactive flies startled by 3 min of incubator light were indistinguishable in experimental animals and parental controls (Figures 5A and 5B). At the restrictive temperature of 31°C, when inactivation of GAL80^ts^ allowed the transcription of *Kir2.1* in experimental flies, basal and light-induced locomotor activity decreased relative to controls (Figures 5A and 5B).

To examine whether the direct stimulation of helicon cells could override the inhibitory effect of dFB neurons and extend waking time, we targeted the light-driven actuator CsChrimson (Klapoetke et al., 2014, Zemelman et al., 2002) under *R24B11-GAL4* control to helicon cells and measured locomotion under closed-loop conditions. The movements of individual flies were continuously monitored and used to trigger three pulses of 630-nm light (3 ms at 20 Hz) after > 3 min of inactivity; the pulse triplet was repeated every 30 s until the next movement occurred (Figure 5C). Control flies lacking the obligatory CsChrimson cofactor all-*trans* retinal, which in adult *Drosophila* must be supplied from external sources (Klapoetke et al., 2014), showed normal levels of sleep despite receiving thousands of light pulses during the 24-hr analysis period (Figures 5D and 5E). The lack of an intrinsically arousing effect of red illumination reflects the minimal intensity of stimulation and the relative insensitivity of *Drosophila*’s photoreceptors at 630 nm (Minke and Kirschfeld, 1979). Rest in retinal-fed flies, in contrast, proved to be sensitive to optical disruption: experimental animals harboring functional CsChrimson in helicon cells maintained elevated levels of activity over a full 24-hr period while being exposed to lower light doses than controls (Figures 5D–5G). Individual behavior, color-encoded in 15-min time bins, illustrates at higher resolution the inverse relationship between locomotor activity and light exposure (Figures 5F and 5G): the more effective the optogenetically evoked helicon cell activity was in keeping an animal awake, the fewer optical stimuli that animal consumed.

### Helicon Cells Excite R2 Ring Neurons

**Figure 6:**
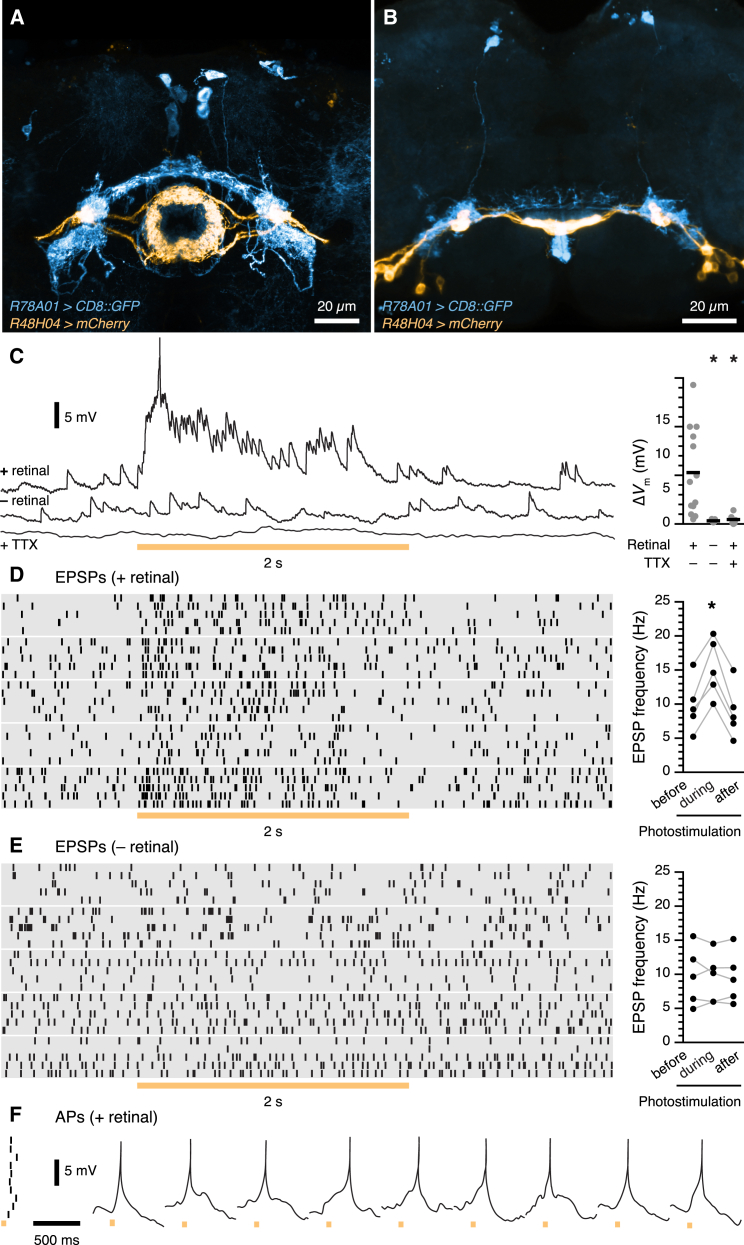
Helicon Cells Excite R2 Neurons of the Ellipsoid Body (A and B) Anterior (A) and dorsal (B) views of helicon cells labeled by *R78A01-GAL4* (blue) and R2 neurons marked by *R48H04-LexA* (yellow). (C) Membrane potentials of R2 neurons during optogenetic stimulation of helicon cell activity (orange bars, 2 s illumination at 630 nm). Top: membrane potential traces (left) and changes in membrane potential baseline (right) in the presence and absence of retinal or in the combined presence of retinal and 1 μM tetrodotoxin (TTX). The lack of retinal or the presence of TTX blocks the optogenetically induced depolarization (n = 6–13 cells per group; p = 0.0002, Kruskal-Wallis ANOVA). Asterisks indicate significant differences from control conditions in pairwise *post hoc* comparisons. (D) EPSP rasters (left) and EPSP frequency modulation (right) of five R2 neurons during five optogenetic stimulation trials of helicon cell activity in retinal-fed flies. One-way repeated-measures ANOVA detects a significant effect of photostimulation (p < 0.0001). (E) EPSP rasters (left) and EPSP frequency modulation (right) of five R2 neurons during five optogenetic stimulation trials of helicon cell activity in vehicle-treated flies. One-way repeated-measures ANOVA fails to detect a significant effect of photostimulation (p = 0.8376). (F) Spike raster (left) and membrane potential (right) of an R2 neuron during optogenetic stimulation of helicon cell activity (orange bars; 50 ms illumination at 630 nm). Each of 9 consecutive light pulses elicits an action potential (AP). See also Figures S3 and S4.

The axonal branches of helicon cells innervate the bulb and concentric rings of the ellipsoid body (Figure 3C), where they lie in close apposition to arborizations of R2 neurons (Figures 6A and 6B). Arrestingly, R2 neurons have been pinpointed as a principal source of sleep pressure (Liu et al., 2016). Prolonged periods of R2 neuron activity are thought to contract a sleep debt that is sensed and cleared by dFB neurons. Sleep homeostasis may thus involve an autoregulatory loop in which dFB and R2 neurons are recurrently connected via helicon cells. If helicon cells provide significant excitation to R2 neurons, their inhibition by dFB neurons—whose output will, in turn, reflect the activity history of R2 neurons (Liu et al., 2016)—could throttle the excitatory drive to R2 neurons, allowing the system to reset during sleep.

Despite their suggestive anatomical proximity in the ellipsoid body (Figures 6A and 6B), there is presently no functional evidence that helicon cells and R2 neurons are indeed connected and that these connections have the excitatory polarity needed to close the recurrent circuit we envisage. To probe for excitatory synapses from helicon cells to R2 neurons, we monitored the membrane potentials of R2 neurons (which were targeted on the basis of their *R48H04-LexA*-driven GFP expression; Figure S4) while photostimulating helicon cells (which expressed CsChrimson under the control of *R78A01-GAL4*, a strong driver recapitulating the *R22H05-GAL4* pattern; Figure S3C). Under basal conditions, R2 neurons were showered by large excitatory postsynaptic potentials (EPSPs) that arrived at widely variable rates averaging 9.44 ± 1.41 Hz (mean ± SEM; range, 0.58–21.58 Hz; n = 5 cells) (Figure 6C). Illumination at 630 nm, sustained for 2 s, elevated the mean EPSP frequency by 62% to 15.33 ± 1.73 Hz (Figure 6D); during these barrages of optogenetically stimulated synaptic input, the membrane potential baseline depolarized by 5.30 ± 1.25 mV (mean ± SEM, n = 5 cells), beyond spike threshold (Figure 6C). Because light-evoked depolarizations vanished in the absence of retinal or the presence of 1 μM tetrodotoxin (Figures 6C and 6E), they were caused by action potential-driven transmission from presynaptic helicon cells. Even single pulses of light could elicit reliable spiking of R2 neurons (Figure 6F), attesting to the powerful influence helicon cells exert over the activity of these postsynaptic partners.

### Helicon Cell Activation Induces Rebound Sleep

**Figure 7:**
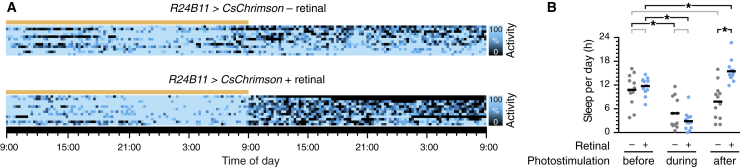
Intense Helicon Cell Activation Induces Rebound Sleep (A) Locomotor activity (blue) of 12 vehicle-treated (top) and 12 retinal-fed flies (bottom) expressing CsChrimson under *R24B11-GAL4* control during 24 hr of photostimulation at 20 Hz and a 24-hr recovery period immediately afterward. Individuals in each group are sorted in descending order of inactivity during the recovery period. Colored squares represent 15-min time bins. Within each bin, the percentage of time spent moving is color-coded according to the look-up table on the right. Stimulation light pulses notwithstanding, the animals were raised and kept in constant darkness. (B) Photostimulation generates rebound sleep in retinal-fed flies expressing CsChrimson under *R24B11-GAL4* control (blue, n = 12 flies) but not in vehicle-treated controls (gray, n = 12 flies) (p < 0.0001, one-way ANOVA). Circles symbolize individual flies; horizontal lines indicate group means. Asterisks indicate significant differences in planned pairwise *post hoc* comparisons (black brackets); gray brackets denote pairwise comparisons without significant differences.

Given the strength of the excitatory connections from helicon cells to R2 neurons, intense helicon cell activity is expected to filter through to R2 neurons and drive the plastic changes thought to represent accumulating sleep pressure, just as prolonged R2 neuron activation does (Liu et al., 2016). In our previous optogenetic stimulation experiments (Figure 5), we activated helicon cells minimally, and only when needed, to occlude the rest-promoting effect of dFB neurons without triggering homeostatic compensation. To test whether more forceful activation of the same neurons could induce a homeostatic response, we maintained helicon cells in an optogenetically induced UP state for 24 hr by delivering 3-ms pulses of stimulation light continually at 20 Hz (Figure 7). Because the necessary light exposures exceeded those for minimal stimulation (Figure 5E) by approximately three orders of magnitude, sleep disruptions of presumably visual origin were now also commonly seen in controls (Figure 7). These sleep disruptions obscured the wake-promoting effect of direct helicon cell stimulation (see Figures 5D and 5G for comparison) but were too mild to initiate rebound sleep on their own (Figures 7A and 7B). After the reconstitution of CsChrimson with all-*trans* retinal, however, which allowed helicon cells to be entrained to the 20-Hz optical stimulus, the sleep control circuitry tipped into rebound mode: experimental flies fell quiescent at the end of photostimulation (Figure 7A) and slept an excess of 7.64 hr relative to controls during the subsequent 24-hr day (Figure 7B).

## Discussion

### Imposing Sleep on an Organism

Sleep, an organismal phenomenon with many physiological and behavioral facets, is controlled by a handful of neurons with narrowly restricted axonal projections (Figure 1A), creating the apparent paradox of a local action with systemic consequences. A solution to this paradox could take several forms. One possibility is that the sleep-promoting cells do not themselves communicate with a wide range of postsynaptic targets but, rather, act indirectly by inhibiting arousal centers, which provide divergence through their own widespread projections. In other words, sleep would be induced by the widely felt withdrawal of a wake-promoting signal, not a diffusely broadcast command to go to sleep. Our data show that sleep-promoting neurons can directly suppress locomotor activity and blunt visual responses (Figure 4). Sleep-control neurons thus impose sleep via efferent circuits that include direct as well as indirect pathways acting through inhibition of arousal. Their combined effects on these circuits must cause the full spectrum of systemic changes associated with sleep.

How many efferent circuits are there, and what are the functional relationships among them? Although the restricted expression of *AstA-R1*^RNAi^ eliminates the electrophysiological response of helicon cells to AstA (Figures 4F and 4G), the same manipulation only incompletely phenocopies the sleep loss seen in homozygous *AstA-R1*^*MB07922*^ mutants (Figures 2A, 3D, 3E, and S2). These results demonstrate a sleep-promoting effect of inhibiting helicon cells, but they also suggest that helicon cells are only one of several dFB outputs used to induce sleep. The potential for specificity in synaptic communication between dFB neurons and their downstream partners raises the possibility that the different behavioral and physiological manifestations of sleep might be separable at the level of dedicated output circuits. In such an arrangement, different efferent channels would gate locomotion or set sensory thresholds, and selective interference with individual channels may dissociate sleep features that are normally grouped.

In an alternative model, neurons of the central complex, and especially the ellipsoid body, may represent a site where a strategically placed gate can enact many sleep-related changes at once. Large amounts of sensory data from different modalities fan into the ellipsoid body, which uses these data to construct representations of visual space and the animal’s position and orientation within it (Green et al., 2017, Heinze and Homberg, 2007, Kim et al., 2017, Seelig and Jayaraman, 2015, Seelig and Jayaraman, 2013). These representations then fan out to inform a range of actions, such as the ability to alternate between flight, walking, and climbing (Harley and Ritzmann, 2010, Ilius et al., 1994); to adjust the speed of locomotion in response to arousing stimuli (Lebestky et al., 2009); to negotiate turns, gaps, and obstacles (Harley and Ritzmann, 2010, Martin et al., 2015, Triphan et al., 2010); and to navigate to memorized locations (Neuser et al., 2008, Ofstad et al., 2011). The arrangement thus resembles an informational bow tie (Csete and Doyle, 2004), with a broad fan of incoming data flowing into a central knot and from there into another broad fan of outgoing motor instructions. In manufacturing, bow tie architectures are advantageous because they allow flexibility in the transformation of raw materials (sensory data) into products (actions) and because they operate economically and efficiently due to the small sizes of their processing cores. However, focused attack on these cores can cause the entire system to shut down. Could sleep-promoting neurons target this vulnerability?

### Balancing Sleep Need and Sleep

**Figure 8:**
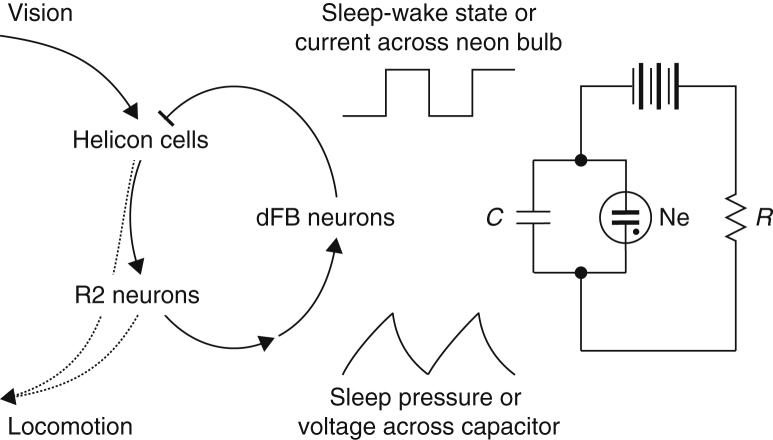
The Sleep Homeostat as a Relaxation Oscillator Helicon cells respond to visual input and play a permissive role in locomotion, either by virtue of their excitatory synapses with R2 neurons or through other pathways. R2 neuron activity generates sleep pressure that is communicated to dFB neurons via currently unidentified synaptic connections or non-synaptic mechanisms. The activation of dFB neurons during sleep inhibits helicon cells and, thus, impedes the flow of visual signals to R2 neurons; this raises the visual sensory threshold, blocks locomotion, and reverses the build-up of sleep pressure due to R2 neuron activity, which is driven, in part, by excitation from helicon cells. Because dFB neurons switch between electrical activity and silence, the sleep homeostat functions as a relaxation oscillator akin to the electrical circuit on the right. Here, a capacitor (*C*) is charged through a resistor (*R*) and discharged through a neon bulb (Ne) when the voltage across the capacitor exceeds the ignition threshold of the bulb. Common to the biological and electrical circuits is the conversion of a continuous process (changes in sleep pressure or voltage) into binary state changes (an organism that is asleep or awake; a bulb that is lit or dark).

Among the postsynaptic partners of helicon cells are R2 ring neurons of the ellipsoid body (Figure 6), whose activity generates sleep pressure that is sensed by the dFB (Liu et al., 2016). The contours of an autoregulatory loop have thus emerged in which sleep-promoting dFB neurons communicate via helicon cells with R2 neurons, and the activity of these ring neurons is relayed back to dFB neurons (Figure 8). We imagine that, as sleep pressure builds during prolonged R2 neuron firing, activity-dependent plasticity (Liu et al., 2016) augments the excitatory drive to dFB neurons or instructs them to step up their intrinsic excitability. As a result, dFB neurons switch to the electrically active state and release inhibition. This pushes helicon cells into the hyperpolarized DOWN state (Figure 4), mutes their spiking, and deprives R2 neurons of a powerful source of excitation (Figures 6 and 7). By virtue of this circular arrangement, dFB-derived inhibition can impose intermittent periods of rest on R2 neurons.

The recurrent R2-dFB-helicon neuron circuit resembles a relaxation oscillator rather than a continuous feedback controller. Continuous feedback would ensure stable operation under a variable load, whereas a relaxation oscillator, such as an electric flasher circuit (Figure 8) or a water clock with a liquid-driven escapement, converts a continuous input signal into a binary output. To this end, the feedback loop contains a switching device that alternates between “fill” and “discharge” modes. A capacitor or reservoir is charged and emptied as its fill level rises to the voltage threshold of a bulb or the opening pressure of a valve (Figure 8). There are unmistakable parallels between these fill and discharge cycles and periods of accumulation and extinction of sleep debt and also between the voltage- or pressure-controlled relief paths of the engineered systems and the switching behavior of dFB neurons, which transition between a silent OFF state when sleep pressure is low (fill mode) and an active ON state when sleep pressure is high (discharge mode) (Donlea et al., 2014, Pimentel et al., 2016). Despite these parallels, many crucial questions remain. They include where precisely along the still unexplored R2-dFB neuron interface sleep debt accrues, in what physical form it is stored, how its accumulation to threshold actuates the dFB switch, and how the accumulated sleep debt is cleared.

## STAR★Methods

### Key Resources Table

**Table:** 

REAGENT or RESOURCE	SOURCE	IDENTIFIER
**Antibodies**		

Anti-GFP (Chicken)	Abcam	ab13970; RRID: AB_300798
Anti-GFP (Mouse)	Memorial Sloan Kettering Monoclonal Antibody Facility	Htz-GFP-19C8
Anti-AstA (Mouse)	Developmental Studies Hybridoma Bank (University of Iowa)	5F10; RRID: AB_528076
Goat anti-chicken, Alexa Fluor 488	Thermo Fisher Scientific	A11039; RRID: AB_2534096
Goat anti-mouse, Alexa Fluor 546	Thermo Fisher Scientific	A11003; RRID: AB_2534071
Streptavidin, Alexa Fluor 568	Thermo Fisher Scientific	S11226; RRID: AB_2315774

**Chemicals, Peptides, and Recombinant Proteins**		

RNaseOUT Recombinant Ribonuclease Inhibitor	Invitrogen (Thermo Fisher Scientific)	10777019
cOmplete Protease Inhibitor Cocktail	Roche	11873580001
IGEPAL CA-630	Sigma	I3021
1,2-dihexanoyl-*sn*-glycero-3-phosphocholine	Avanti Polar Lipids	850305
Protein G Mag Sepharose beads	GE Healthcare	28944008
PicoPURE RNA Isolation Kit	Invitrogen (Thermo Fisher Scientific)	KIT0204
RNA 6000 Pico Kit	Agilent	5067-1513
SMART-Seq v4 Ultra Low Input RNA Kit for Sequencing	Clontech	634890
Agencourt AMPure XP system	Beckman Coulter	A63881
LightCycler 480 Probes Master	Roche	04707494001
PFA	Electron Microscopy	15700
Normal goat serum	Sigma	G9023
AstA (SRPYSFGL-NH_2_)	PeptideSynthetics	N/A
Scrambled AstA (GRFSSYLP-NH_2_)	PeptideSynthetics	N/A
Tetrodotoxin citrate	Tocris	1069
All-trans retinal	Sigma	R2500
Vectashield antifade mounting medium	Vector Laboratories	H-1000

**Experimental Models: Organisms/Strains**		

*Drosophila*: *w*^*1118*^	Bloomington *Drosophila* Stock Center (BDSC)	RRID: BDSC_3605
*Drosophila*: *Canton-S*	BDSC	RRID: BDSC_64349
*Drosophila*: *w*^*1118*^*;+;R23E10-GAL4*	BDSC	RRID: BDSC_49032
*Drosophila*: *w*^*1118*^*; UAS-DenMark, UAS-*^*GFP*^*DSyd-1*	David Owald	N/A
*Drosophila*: *w*^*1118*^*;+;AstA*^*MB10261*^	BDSC	RRID: BDSC_29107
*Drosophila*: *w*^*1118*^*; UAS-AstA*^*RNAi*^	Vienna *Drosophila* Resource Center (VDRC)	103215KK
*Drosophila*: *w*^*1118*^*; UAS-Mip*^*RNAi*^	VDRC	106076KK
*Drosophila*: *w*^*1118*^*; UAS-AstA*	This paper	N/A
*Drosophila*: *w*^*1118*^*; UAS-EGFP::mL10a*	F. Rob Jackson	N/A
*Drosophila*: *AstA-R1*^*MB07922*^	BDSC	RRID: BDSC_25577
*Drosophila*: *elav-GAL4[C155]*	BDSC	RRID: BDSC_458
*Drosophila*: *w*^*1118*^*; UAS-AstA-R1*^*RNAi*^	VDRC	101395KK
*Drosophila*: *w*^*1118*^*; +; R22H05-GAL4*	BDSC	RRID: BDSC_49002
*Drosophila*: *w*^*1118*^*; +; R22H10-GAL4*	BDSC	RRID: BDSC_49005
*Drosophila*: *w*^*1118*^*; +; R24B11-GAL4*	BDSC	RRID: BDSC_49070
*Drosophila*: *w*^*1118*^*; R23E10-LexA*	BDSC	RRID: BDSC_52693
*Drosophila*: *w*^*1118*^*; UAS-CD8::GFP*	BDSC	RRID: BDSC_32186
*Drosophila*: *w*^*1118*^*; +; lexAop-CD4::mCherry*	Scott Waddell	N/A
*Drosophila*: *w*^*1118*^*; lexAop-P2X2*	This paper	N/A
*Drosophila*: *w*^*1118*^*; UAS-Kir2.1::GFP*	Evan Harrell and Gero Miesenböck	N/A
*Drosophila*: *w*^*1118*^*; +; tubP-GAL80*^*ts*^	BDSC	RRID: BDSC_7017
*Drosophila*: *w*^*1118*^*; UAS-CsChrimson*	BDSC	RRID: BDSC_55135
*Drosophila*: *w*^*1118*^*; +; R78A01-GAL4*	BDSC	RRID: BDSC_39985
*Drosophila*: *w*^*1118*^*; R48H04-LexA*	BDSC	RRID: BDSC_53609
*Drosophila*: *w*^*1118*^*; +; R58H05-GAL4*	BDSC	RRID: BDSC_39198
*Drosophila*: *w*^*1118*^*; R24B11-LexA*	BDSC	RRID: BDSC_53547

**Software and Algorithms**		

Sleep analysis from Trikinetics activity counts	Paul Shaw	N/A
Software for optogenetic stimulation and movement detection	This paper	N/A

### Contact for Reagent and Resource Sharing

Further information and requests for resources and reagents should be directed to and will be fulfilled by the Lead Contact, Gero Miesenböck (gero.miesenboeck@cncb.ox.ac.uk).

### Experimental Model and Subject Details

*Drosophila melanogaster* strains were grown on media of sucrose, yeast, molasses, and agar, and maintained on a 12 h light:12 h dark schedule at 25°C unless they expressed GAL80^ts^ (McGuire et al., 2003); in this case the experimental animals and all relevant controls were grown at 18°C. Flies expressing CsChrimson were transferred to food supplemented with 2 mM all-*trans* retinal in DMSO upon eclosion and reared in darkness thereafter. All studies were performed on male and/or female animals, as indicated below, aged 4–10 days at the beginning of the analysis period.

The *AstA*^*MB10261*^ and *AstA-R1*^*MB07922*^ mutants carry insertions of MiMIC cassettes (Venken et al., 2011). Driver lines *R23E10-GAL4*, *R23E10-LexA*, and *104y-GAL4* were used to direct transgene expression to dFB neurons (Jenett et al., 2012, Rodan et al., 2002); *R22H05-GAL4*, *R22H10-GAL4*, *R24B11-GAL4*, and *R78A01-GAL4* were used to target helicon cells (Jenett et al., 2012); *R58H05-GAL4* and *R48H04-LexA* provided access to R2 neurons of the ellipsoid body (Jenett et al., 2012). Effector transgenes encoded membrane-bound fluorescent proteins (*UAS-CD8::GFP*; *lexAop-mCherry*); the dendritic and presynaptic markers DenMark (Nicolaï et al., 2010) and ^GFP^DSyd-1 (Owald et al., 2010), respectively; a GFP-tagged version of the ribosomal protein mL10a for the cell-specific analysis of polysome-bound transcripts (Huang et al., 2013); the ion channels Kir2.1 (Baines et al., 2001) or P2X2 (Lima and Miesenböck, 2005); the optogenetic actuator CsChrimson (Klapoetke et al., 2014); or hairpin constructs for RNA-mediated interference with the expression of *AstA* (transformant 113215KK), its receptor *AstA-R1* (transformant 101395KK), and *Mip* (transformant 106076KK) (Dietzl et al., 2007). The *UAS-AstA* transgene incorporates a codon-optimized synthetic cDNA sequence of 474 bp (Eurofins MWG Operon) in pUAST (Brand and Perrimon, 1993) and was integrated into the genome at a random location (Rainbow Transgenics).

### Method Details

#### Behavior

##### Sleep Measurements

Female flies were individually inserted into 65-mm glass tubes, loaded into Trikinetics *Drosophila* Activity Monitors, and housed under 12 h light:12 h dark schedules. Periods of inactivity lasting at least 5 min were classified as sleep. Mechanical sleep deprivation used the SNAP method for 12 h overnight (Shaw et al., 2002). Sleep lost and regained was calculated for each fly by using the 24-h period preceding deprivation as the baseline. Visual arousal thresholds were estimated by exposing flies every 2 h to a 3-min pulse of incubator light and determining the percentage of sleeping flies awakened.

##### Circadian Analysis

Male flies were housed individually in 65-mm glass tubes containing 4% sucrose, 2% agar medium. Locomotor activity was measured in Trikinetics *Drosophila* Activity Monitors for 10 days in constant darkness. Rhythmicity and period length were analyzed using χ^2^ tests in the ActogramJ plugin (Schmid et al., 2011) for ImageJ (NIH).

##### Open- and Closed-Loop Optogenetics

Female flies were individually inserted into 65-mm glass tubes and loaded into a custom-built array of light-tight chambers, which were each equipped with a high-power LED (Multicomp OSW-4388, 630 nm). The apparatus was operated in a temperature-controlled incubator (Sanyo MIR-154) at 25°C. For movement tracking, the chambers were continuously illuminated from below using low power infrared (850 nm) LEDs and imaged from above with a high-resolution CMOS camera (Thorlabs DCC1545M), using an 8-mm lens (Thorlabs MVL8M23) and a long-pass filter (Thorlabs, FEL800nm) to reject photostimulation light. A virtual instrument written in LabVIEW 9 (National Instruments) extracted real-time position data from video images by subtracting the most recently acquired image from a temporally low-pass filtered background. Non-zero pixels in the difference image indicated that a movement had occurred, with the centroid of the largest cluster of non-zero pixels taken to represent the fly’s new position. To eliminate noise, intensity and size thresholds were applied to pixel clusters in the difference image, and movements < 2.5 mm were discarded. If no movement was detected, the fly was assumed to have remained at its last known location.

Open-loop stimulation used a continuous train of 3-ms light pulses (∼28 mW/cm^2^) at 20 Hz. Under closed-loop conditions, blocks of 3 stimulation light pulses (3 ms duration, 20 Hz, ∼28 mW/cm^2^) were triggered after 3 min of inactivity and repeated every 30 s until the next movement occurred. Periods of inactivity lasting at least 5 min were classified as sleep.

#### Translating Ribosome Affinity Purification

For each biological replicate, the heads of 400 female flies expressing *UAS-EGFP::mL10a* (Huang et al., 2013) in dFB neurons were collected at 4–6 days post-eclosion and homogenized in 500 μl of extraction buffer (pH 7.3) containing 20 mM HEPES, 150 mM KCl, 5 mM MgCl_2_, 0.5 mM dithiothreitol, 100 μg/ml cycloheximide, 100 U/ml RNaseOUT (Invitrogen), and 1 × cOmplete Protease Inhibitor (Roche). Lysates were centrifuged at 20,000 *g* for 20 min at 4°C. The supernatants were incubated with 1/8 volume of 10% (v/v) Igepal CA-630 and 1/8 volume of 300 mM 1,2-dihexanoyl-*sn*-glycero-3-phosphocholine (Avanti Polar Lipids) for 5 min on ice and were then applied to 25 μl Protein G Mag Sepharose beads (GE Healthcare) coated with mouse monoclonal anti-GFP antibody (Htz-GFP-19C8, Memorial Sloan Kettering Monoclonal Antibody Facility). After incubation for 1 h at 4°C, the beads were washed 5 times with 500 μl of wash buffer (pH 7.3) containing 20 mM HEPES, 350 mM KCl, 5 mM MgCl_2_, 0.5 mM dithiothreitol, 100 μg/ml cycloheximide, 1% (v/v) Igepal CA-630, and 40 U/ml RNaseOUT.

#### Reverse Transcription and Quantitative Real-Time PCR

RNA was isolated from immunoprecipitated ribosomes using the PicoPURE RNA Isolation Kit (Life Technologies) and quantitated with the help of an RNA 6000 Pico Kit (Agilent) on an Agilent Bioanalyzer. Five ng of RNA were reverse-transcribed into cDNA and amplified using the SMART-Seq v4 Ultra Low Input RNA Kit for Sequencing (Clontech). The resulting cDNA was purified using Agencourt AMPure XP beads (Beckman Coulter). Transcript levels were determined by quantitative real-time PCR on a LightCycler 480 system (Roche) using LightCycler 480 Probes Master (Roche) in 5 μl reactions containing 400 nM of each gene-specific primer and ∼5 ng of pre-amplified cDNA. All samples were run in technical triplicates. Relative transcript levels were estimated with the help of the 2^-ΔΔ*C*t^ method (Livak and Schmittgen, 2001), using the geometric mean of the *C*_t_ values of three housekeeping genes (*Cyp1*, *Ef1a100E* and *Rap2l*) for normalization.

#### Electrophysiology

Male and female flies with a dorsal cranial window were head-fixed to a custom mount and placed on a spherical treadmill (Buchner, 1976, Seelig et al., 2010, Pimentel et al., 2016). The treadmill consisted of an air-supported trackball made of extruded styrofoam (13 mm diameter; 50 mg) in a 14 mm tube. An image of a small region of the ball’s surface under 640 nm LED illumination was relayed onto the sensor of an optical mouse (Logitech M-U0017). The sensor was interfaced with a microcontroller board (Arduino Due) based on the Atmel SAM3X CPU and read out in real time using the onboard D/A converter. The resolution of the readout corresponds to 4 mm/s increments in the tangential speed of the trackball.

The brain was continuously superfused with extracellular solution equilibrated with 95% O_2_/5% CO_2_ and containing 103 mM NaCl, 3 mM KCl, 5 mM TES, 8 mM trehalose, 10 mM glucose, 7 mM sucrose, 26 mM NaHCO_3_, 1 mM NaH_2_PO_4_, 1.5 mM CaCl_2_, 4 mM MgCl_2_, pH 7.3. Somata of CD8::GFP-labeled helicon cells or R2 neurons were visually targeted with borosilicate glass electrodes (7-13 MΩ). The internal solution contained 140 mM potassium aspartate, 10 mM HEPES, 1 mM KCl, 4 mM MgATP, 0.5 mM Na_3_GTP, 1 mM EGTA, pH 7.3. Signals were acquired with a Multiclamp 700B amplifier (Molecular Devices), filtered at 6–10 kHz, and digitized at 10–20 kHz using an ITC-18 data acquisition board (InstruTECH) controlled by the Nclamp/NeuroMatic package. Data were analyzed using NeuroMatic software (http://www.neuromatic.thinkrandom.com) and custom procedures in Igor Pro (Wavemetrics).

For applications of peptides to the FB layer innervated by sleep-control neurons, glass electrodes were filled with 3 mM synthetic AstA (SRPYSFGL-NH_2_) in extracellular solution or a control peptide containing the same amino acids in a scrambled sequence (GRFSSYLP-NH_2_). The electrodes were visually guided to the central complex, using GFP-positive neurites as landmarks. The application of a 250 ms pressure pulse (68 kPa; Picospritzer III) resulted in the ejection of ∼40 pl of solution.

For genetically targeted stimulation of dFB neurons expressing P2X2, a glass electrode containing 500 μM ATP in extracellular solution was positioned unilaterally in the region housing the dendritic fields of these neurons. During periods of stimulation, a 500 ms pressure pulse (68 kPa; Picospritzer III) was applied every 3 s.

#### Confocal Microscopy

Brains were dissected in PBS (1.86 mM NaH_2_PO_4_, 8.41 mM Na_2_HPO_4_, 175 mM NaCl) and fixed for 30–45 min in 4% (w/v) paraformaldehyde in PBS at 4°C. For immunostaining, brains were incubated in primary antibodies for 48 h (1:1,000 chicken anti-GFP, Abcam; 1:2 mouse anti-AstA, Developmental Studies Hybridoma Bank, University of Iowa), followed by secondary antibodies for 24 h (1:1,000 anti-chicken antibody conjugated to Alexa Fluor 488; 1:1,000 anti-mouse antibody conjugated to Alexa Fluor 546; both from Invitrogen). Brains containing biocytin-filled neurons were incubated in 1:200 streptavidin conjugated to Alexa Fluor 568 (Invitrogen) in PBS with 0.1% (v/v) Triton X-100 for 48 h. All specimens were mounted in Vectashield (Vector Labs) and imaged on a Leica TCS SP5 confocal microscope.

### Quantification and Statistical Analysis

Data were analyzed in Prism 6 (GraphPad). Group means were compared by one-way or two-way ANOVA, using repeated-measures designs where appropriate, followed by planned pairwise *post hoc* analyses using Holm-Šídák’s multiple comparisons test. Where the assumptions of normality or sphericity were violated (as indicated by Shapiro-Wilk and Brown-Forsythe tests, respectively), group means were compared by Mann-Whitney or Kruskal-Wallis tests, the latter followed by Dunn’s multiple comparisons test. Details of statistical analyses are found in figure legends.

### Data and Software Availability

Requests for raw data and instrumentation and analysis code should be directed to the Lead Contact, Gero Miesenböck (gero.miesenboeck@cncb.ox.ac.uk).

## Supplemental Information

Document S1. Figures S1–S4 and Table S1

Document S2. Article plus Supplemental Information
