# Supplementary material for: Recurrent Circuitry for Balancing Sleep Need and Sleep
Source: Neuron. 2018 Jan 17;97(2):378–389.e4. doi: 10.1016/j.neuron.2017.12.016 (PMC5779612; doi:10.1016/j.neuron.2017.12.016)
Supplement: Document S1. Figures S1–S4 and Table S1 [file mmc1.pdf]

Neuron, Volume *97*

Supplemental Information

Recurrent Circuitry

for Balancing Sleep Need and Sleep

Jeffrey M. Donlea, Diogo Pimentel, Clifford B. Talbot, Anissa Kempf, Jaison J. Omoto, Volker Hartenstein, and Gero Miesenböck

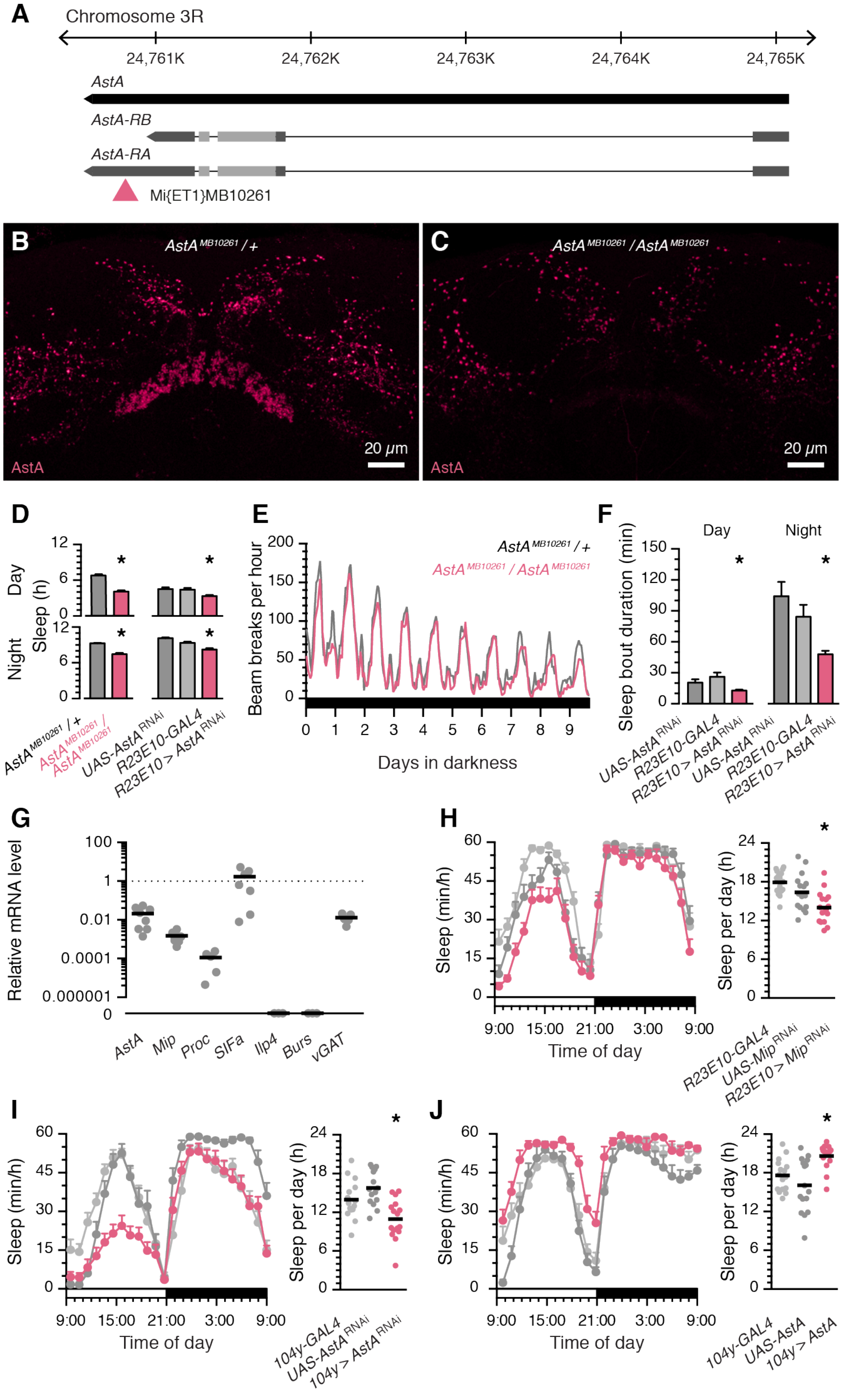

Figure S1, Related to Figure 1. AstA Functions in dFB Neurons to Regulate Sleep
(A) The *AstA* locus encodes two annotated transcripts. The transposon insertion in $AstA^{MB10261}$ disrupts the *AstA-RA* isoform.
(B, C) Immunostaining for AstA in the dFB of homozygous $AstA^{MB10261}$ mutants (C) and heterozygous controls (B).
(D) Homozygous $AstA^{MB10261}$ mutants (red in left panel, mean ± SEM, n = 90–118 flies per group) and flies expressing $AstA^{RNAi}$ under the control of *R23E10-GAL4* (red in right panel, mean ± SEM, n = 99–111 flies per group) sleep less during the day (top left: $p < 0.0001$, Mann-Whitney test; top

right: $p = 0.0050$, Kruskal-Wallis ANOVA) and night (bottom left: $p < 0.0001$, Mann-Whitney test; bottom right: $p < 0.0001$, Kruskal-Wallis ANOVA) than heterozygous *AstA*^{MB10261} mutants or parental controls, respectively. Asterisks on the right denote significant differences from both parental controls in pairwise post-hoc comparisons.
(E) Circadian locomotor rhythmicity is unchanged in homozygous *AstA*^{MB10261} mutants (red; 38/39 flies rhythmic; $\tau = 23.76 \pm 0.04$ hours, mean ± SEM) compared to heterozygous controls (gray; 42/42 flies rhythmic, $\tau = 23.73 \pm 0.04$ hours, mean ± SEM; $p = 0.5983$, t test).
(F) Flies expressing *AstA*^{RNAi} under the control of *R23E10-GAL4* (red) exhibit shorter sleep bouts than parental controls (gray) during the day (left, mean ± SEM; $p < 0.0001$, Kruskal-Wallis ANOVA) and night (right, mean ± SEM; $p < 0.0001$, Kruskal-Wallis ANOVA).
(G) Levels of polysome-bound neuropeptide and GABA transporter transcripts in dFB neurons expressing EGFP::mL10a under the control of *R23E10-GAL4*, relative to the geometric mean of three marker gene transcripts (*Cyp1, Ef1a100E*, and *Rap2l*). Circles denote biological replicates; horizontal lines indicate group means.
(H) Expression of *Mip*^{RNAi} under the control of *R23E10-GAL4* (red) reduces sleep compared to parental controls (light gray: *R23E10-GAL4*/+; dark gray: *UAS-Mip*^{RNAi}/+) (mean ± SEM, n = 16 flies per group). White and black bars denote periods of light and darkness, respectively. Two-way repeated-measures ANOVA of the hour-by-hour sleep timecourse detects a significant genotype × time interaction (left; $p < 0.0001$); one-way ANOVA detects a significant genotype effect on total sleep time (right; $p < 0.0001$). Circles symbolize individual flies; horizontal lines indicate group means. The asterisk denotes a significant difference from both parental controls in pairwise post-hoc comparisons.
(I) Expression of *AstA*^{RNAi} under the control of *104y-GAL4* (red) reduces sleep compared to parental controls (light gray: *104y-GAL4*/+; dark gray: *UAS-AstA*^{RNAi}/+) (mean ± SEM, n = 15–16 flies per group). White and black bars denote periods of light and darkness, respectively. Two-way repeated-measures ANOVA of the hour-by-hour sleep timecourse detects a significant genotype × time interaction (left; $p < 0.0001$); one-way ANOVA detects a significant genotype effect on total sleep time (right; $p = 0.0002$). Circles symbolize individual flies; horizontal lines indicate group means. The asterisk denotes a significant difference from both parental controls in pairwise post-hoc comparisons.
(J) Overexpression of AstA under the control of *104y-GAL4* (red) increases sleep compared to parental controls (light gray: *104y-GAL4*/+; dark gray: *UAS-AstA*/+) (mean ± SEM, n = 16 flies per group). White and black bars denote periods of light and darkness, respectively. Two-way repeated-measures ANOVA of the hour-by-hour sleep timecourse detects a significant genotype × time interaction (left; $p < 0.0001$); Kruskal-Wallis ANOVA detects a significant genotype effect on total sleep time (right; $p = 0.0004$). Circles symbolize individual flies; horizontal lines indicate group means. The asterisk denotes a significant difference from both parental controls in pairwise post-hoc comparisons.
See also Table S1.

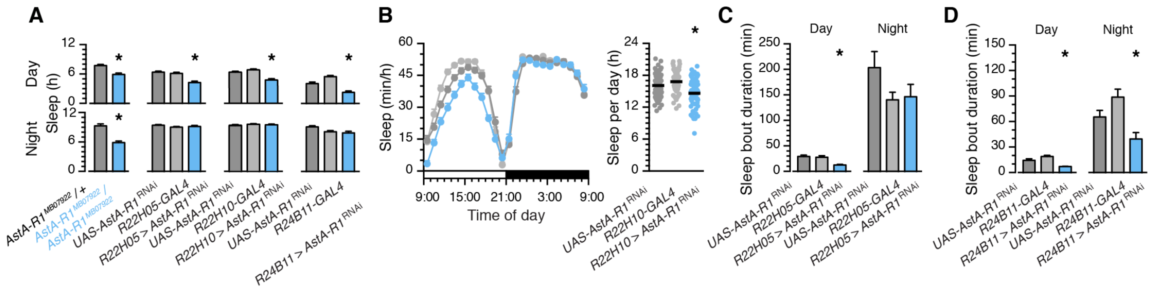

Figure S2, Related to Figure 3. AstA-R1 Functions in Helicon Cells to Regulate Sleep
(A) Homozygous *AstA-R1*^{MB07922} mutants (blue in leftmost panel, mean ± SEM, n = 46–48 flies per group) sleep less than heterozygous control during the day ($p < 0.0001$, Mann-Whitney test) and night ($p < 0.0001$, Mann-Whitney test). Flies expressing *AstA-R1*^{RNAi} under the control of *R22H05-GAL4* (blue in second panel from left, mean ± SEM, n = 63–64 flies per group), *R22H10-GAL4* (blue in third panel from left, mean ± SEM, n = 63–64 flies per group), or *R24B11-GAL4* (blue in rightmost panel, mean ± SEM, n = 30–32 flies per group) sleep less than parental controls during the day ($p < 0.0001$, Kruskal-Wallis ANOVA) but not during the night ($p > 0.1555$, Kruskal-Wallis ANOVA). Asterisks denote significant differences from both parental controls in pairwise post-hoc comparisons.
(B) Expression of *AstA-R1*^{RNAi} under the control of *R22H10-GAL4* (blue) reduces sleep compared to parental controls (light gray: *R22H10-GAL4*/+; dark gray: *UAS-AstA-R1*^{RNAi}/+) (mean ± SEM, n = 63–64 flies per group). White and black bars denote periods of light and darkness, respectively. Two-way repeated-measures ANOVA of the hour-by-hour sleep timecourse detects a significant genotype × time interaction (left; $p < 0.0001$); Kruskal-Wallis ANOVA detects a significant genotype effect on total sleep time (right; $p < 0.0001$). Circles symbolize individual flies; horizontal lines indicate group means. The asterisk denotes a significant difference from both parental controls in pairwise post-hoc comparisons.
(C) Flies expressing *AstA-R1*^{RNAi} under the control of *R22H05-GAL4* (blue) exhibit shorter sleep bouts than parental controls (gray) during the day (left, mean ± SEM; $p < 0.0001$, Kruskal-Wallis ANOVA) but not during the night (right, mean ± SEM; $p = 0.1937$, Kruskal-Wallis ANOVA).
(D) Flies expressing *AstA-R1*^{RNAi} under the control of *R24B11-GAL4* (blue) exhibit shorter sleep bouts than parental controls (gray) during the day (left, mean ± SEM; $p < 0.0001$, Kruskal-Wallis ANOVA) and night (right, mean ± SEM; $p < 0.0001$, Kruskal-Wallis ANOVA). Asterisks denote significant differences from both parental controls in pairwise post-hoc comparisons.

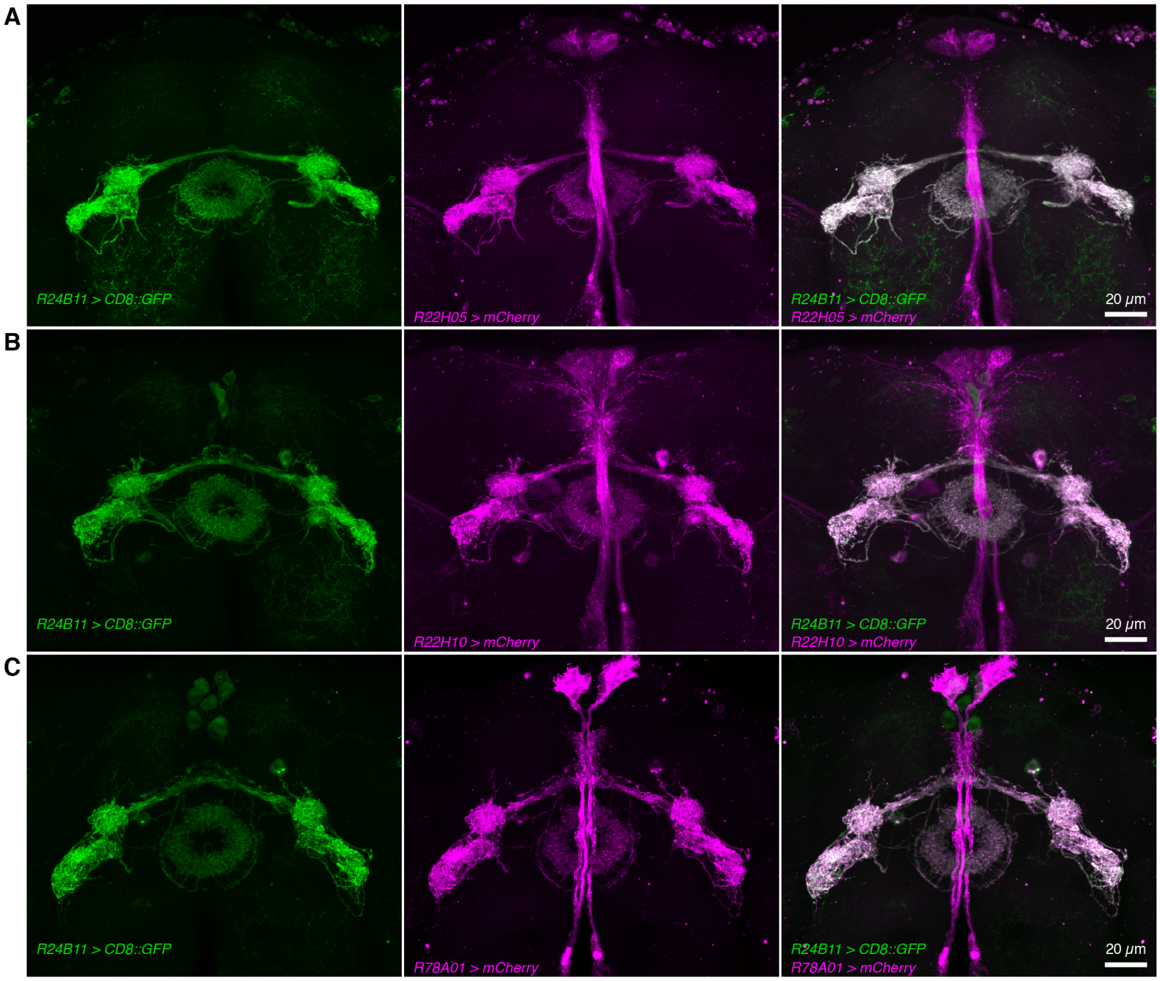

Figure S3, Related to Figures 3 and 6. Genetic Access to Helicon Cells
(A-C) *R24B11-LexA* driven CD8::GFP expression overlaps with the expression of mCherry under the control of *R22H05-GAL4* (A), *R22H10-GAL4* (B), and *R78A01-GAL4* (C) in all four helicon cells, but not in neuroendocrine cells of the pars intercerebralis.

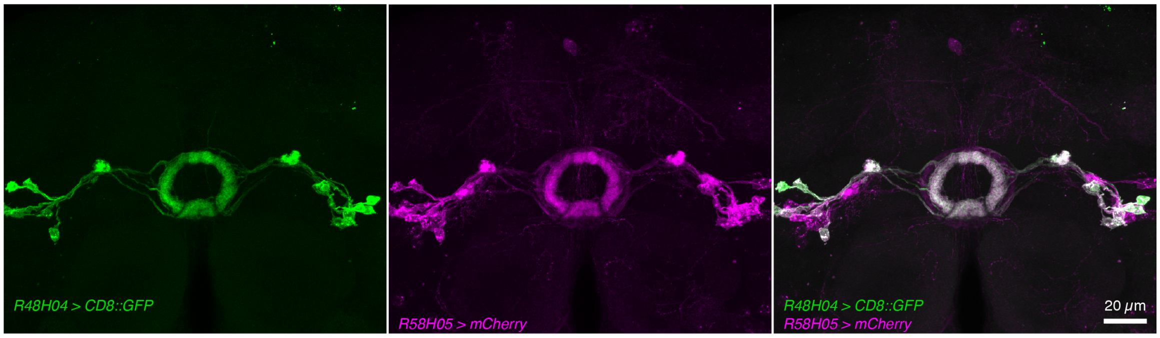

Figure S4, Related to Figure 6. Genetic Access to R2 Neurons
R48H04-LexA drives CD8::GFP expression in ~16 of the 32 R2 neurons expressing mCherry under the control of *R58H05-GAL4* (Liu et al., 2016).

Table S1, Related to Figure S1. Primers for Quantitative Real-Time PCR

Gene	Forward primer (5' - 3')	Reverse primer (5' - 3')
Cyp1	TCGGCAGCGGCATTTCAGAT	TGCACGCTGACGAAGCTAGG
Ef1a100E	ATCAGCTCCGAGGATGACGC	GCCGAGACAGACGTTCCAGA
Rap2l	ACTTCCGTGCATTACGTGCG	CCGACCCGAGCACAACAACT
AstA	GCATTTGGAATTCGCTCAG	GCGTGAAGGGAGTTCATTGT
Mip	AAATCGCGAGGAGATATATAGTCAG	CTGGCCACCAAATTACCG
Proc	CTCCATCGAAAAACACAAACC	CACCTGTGTCCACTTCCACA
SIFa	ACTCTGCTCCTGGTCACGAT	TTTGGCGCTGTCGTAGTCT
Ilp4	GGCACTGGATGTGATTTGTG	CGTTTCCTGTTCAATGTCCTC
Burs	CGCTGTGCCAGTTATATCCA	CTTTGGGACAGAATAGCGAGA
vGAT	AAACGGACGGCTTTAGGC	GGAATTCGTCGATTTTGCAG
